# Case report: Wallerian degeneration: The innate-immune response to adult-onset Still's disease peripheral nerve injury

**DOI:** 10.3389/fneur.2022.1016393

**Published:** 2022-10-19

**Authors:** Hao Cheng, He Lv, Yan Wang, Gui-Qiang Wang

**Affiliations:** ^1^Department of Infectious Diseases and the Center for Liver Diseases, Peking University First Hospital, Beijing, China; ^2^Department of Neurology, Peking University First Hospital, Beijing, China

**Keywords:** adult-onset Still's disease, sensory neuropathy, sensory ganglionopathy, Wallerian degeneration, delayed aggravation, innate-immune response

## Abstract

Peripheral nerve injury is one of the rare complications of adult-onset Still's disease (AOSD). We report a 20-year-old woman diagnosed with AOSD combined with severe sensory neuropathy. She presented with a sore throat, joint pain, rash, and lymphadenopathy. After receiving glucocorticoid therapy, her fever, rash, and inflammatory markers improved. Unexpectedly, 3 weeks after the onset, she experienced sudden paresthesia in her extremities, decreased muscle strength, and diminished tendon reflexes. The electrophysiological examination and peripheral nerve biopsy confirmed immune-mediated severe sensory neuropathy. For the first time, we report typical Wallerian degeneration in AOSD patients with sensory neuropathy by nerve biopsy. Compared with other common symptoms, the delayed aggravation of neurological symptoms may be an important characteristic of sensory neuropathy secondary to AOSD. We emphasize that intensive attention to neurological symptoms after general symptoms control, administration of adequate and appropriate prolonged immunosuppressive therapy, and long-term follow-up are essential for these patients.

## Introduction

Peripheral nerve injury is a rare complication of adult-Onset Still's Disease (AOSD). Wallerian degeneration was observed in distal myelinated fibers, which may cause the delayed aggravation of neurological symptoms during glucocorticoid treatment in an AOSD patient.

The rapidly progressive clinical deterioration of the AOSD patient presented here posed a diagnostic challenge requiring an extensive diagnostic workup. In the literature, only a few case reports describe the main manifestations of AOSD with sensory neuropathy, while most were short of pathological evidence. For the first time, we report typical Wallerian degeneration in AOSD patients with severe sensory neuropathy by nerve biopsy. Careful distinction and prompt diagnosis are critical for adherence to immunosuppressive therapy.

## Case description

**Figure 1:**
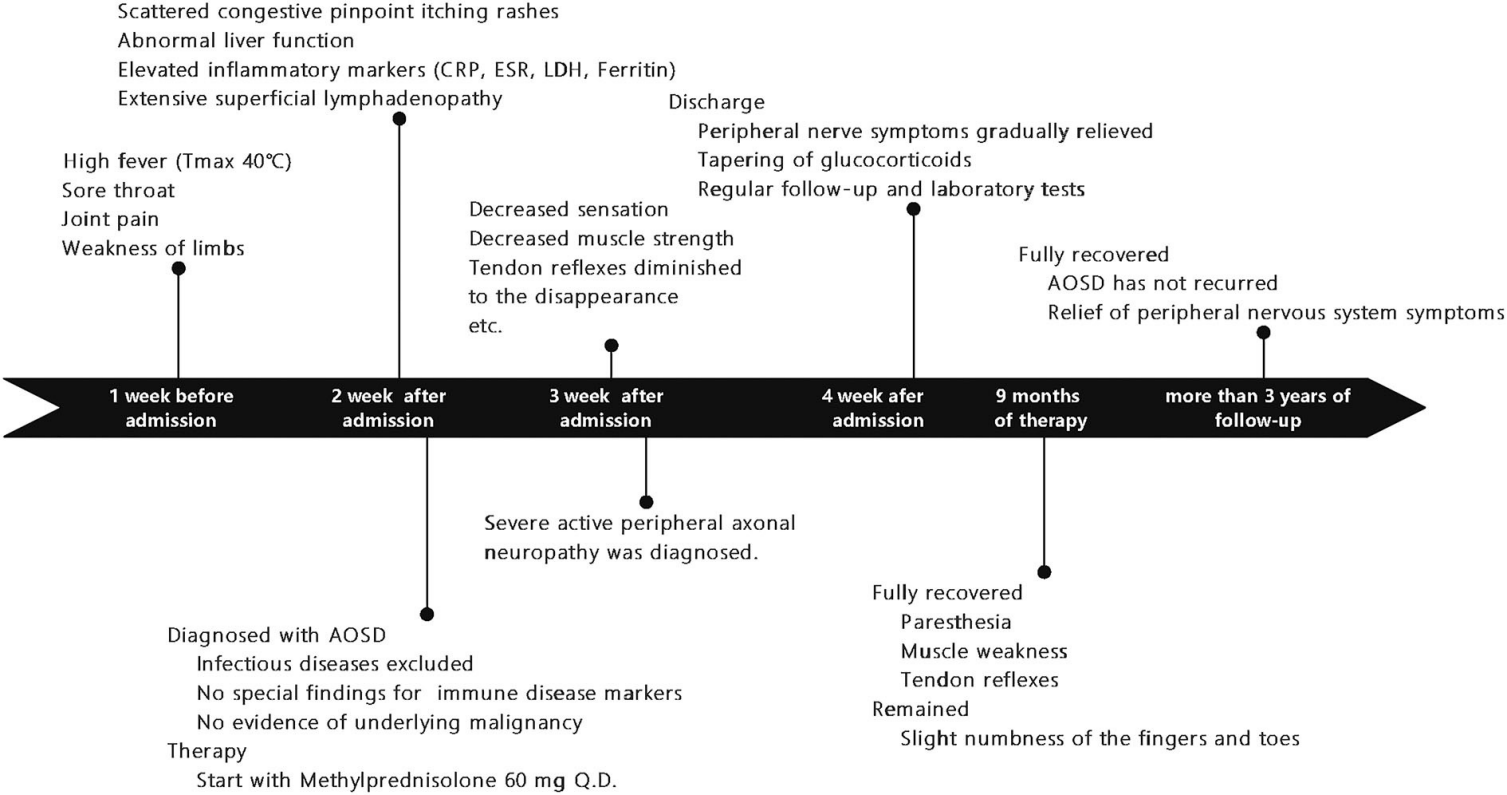
A timeline showing symptoms and exams/interventions performed on the patient. AOSD, Adult-onset Still's disease; CRP, C-reactive protein; LDH, lactate dehydrogenase, ESR, erythrocyte sedimentation rate.

A 20-year-old woman was presented to the emergency clinic with fever and sore throat for one week and joint pain, weakness in limbs, and slight numbness of fingertips and tongue. She denied any tobacco or alcohol (illicit drugs) use, and her family history was not notable. After admission, she continued to experience a high fever (*T*_max_ 40°C). One week after her admission, scattered congestive pinpoint itching rashes appeared on her chest, abdomen, back, and limbs, without ulceration or scaling, accompanied by liver function abnormalities (alanine aminotransferase, ALT_max_ 227 IU/L, reference range of 7–40 IU/L; aspartate transaminase, AST_max_ 193 IU/L, reference range of 13–35 IU/L). The rash was noticeable when the body temperature rose and faded when the body temperature dropped. Inflammatory markers such as serum ferritin (max to >7,500 ng/ml, reference range 11–306.8 ng/ml), erythrocyte sedimentation rate (ESR, max to 50 mm/h, reference range 0–20 mm/h), C-reactive protein (CRP, max to 104 mg/L, normal <5 mg/L), and lactate dehydrogenase (LDH, max to 900 IU/L, reference range 100–240 IU/L) were all significantly elevated. The lymph node ultrasound revealed extensive enlarged superficial lymph nodes (Figure 1).

The clinical diagnosis of AOSD is generally reached by exclusion while investigating a patient with a fever of unknown origin. We carried out detailed screenings for infectious diseases, autoimmune diseases, and tumors. Laboratory examinations for common pathogens (respiratory pathogens, such as influenza virus, mycoplasma, chlamydia, legionella, adenovirus, etc.) and specific disease-related pathogens (cytomegalovirus/Epstein-Barr virus, mycobacterium tuberculosis, rickettsia of typhus*, Coxiella burnetii* of Q fever, Salmonella typhi, Brucella, etc.) were all negative. The blood culture was repeatedly negative, and screening tests for AIDS, hepatitis B, hepatitis C, and syphilis were negative. Antibodies that indicate some autoimmune diseases, such as the anti-double-stranded DNA antibody, antineutrophil cytoplasmic antibodies (ANCA), anti-extractable nuclear antigen (ENA) antibodies, immunoglobulins, protein electrophoresis, immunofixation electrophoresis, complements, rheumatoid factor were all no meaningful found. No significant findings were found in tumor markers, bone marrow biopsy, and imaging examinations of underlying malignant tumors. According to the Yamaguchi criteria (1), the patient was diagnosed with AOSD.

**Figure 2:**
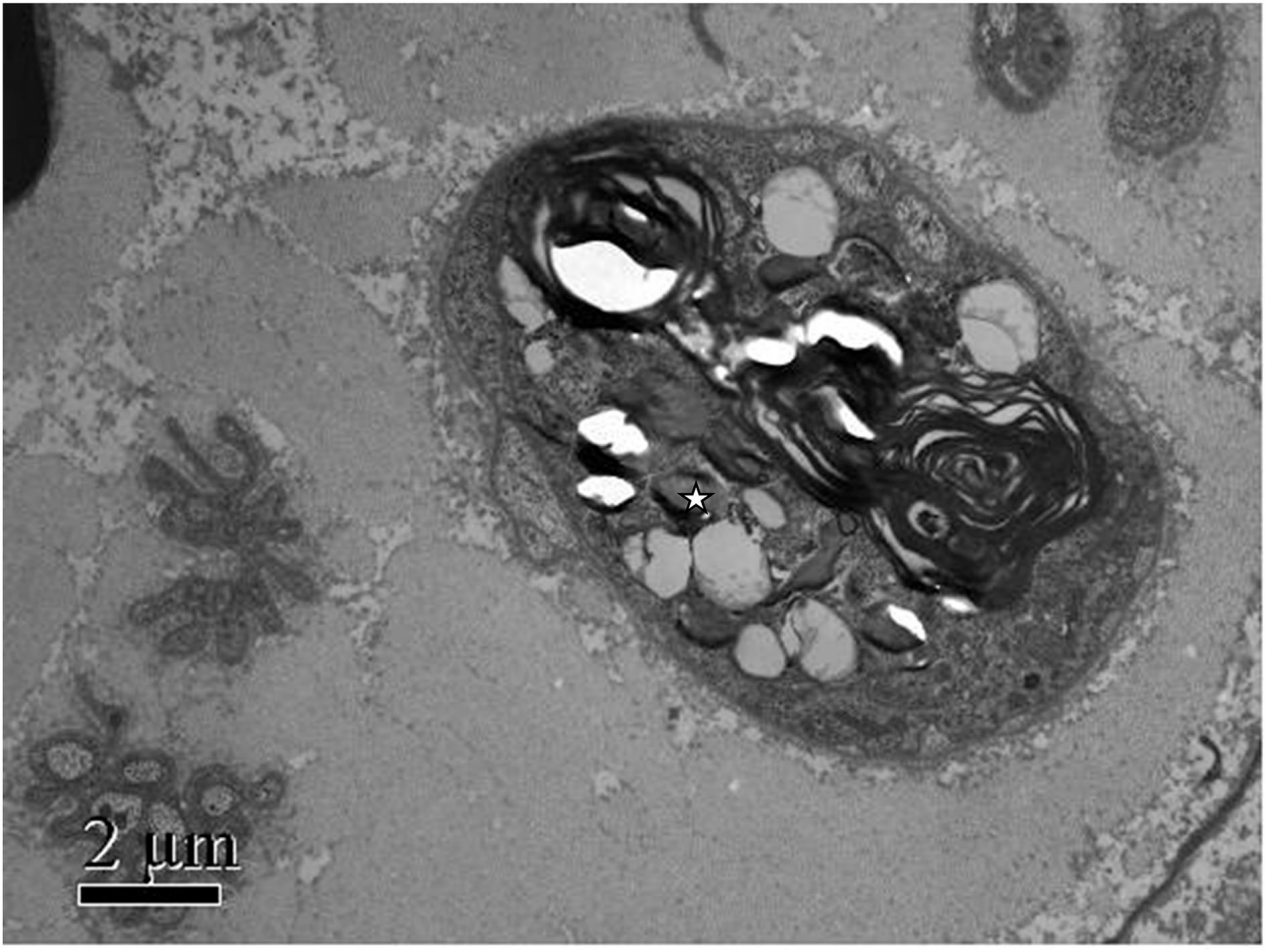
Patient's photograph of the sural nerve biopsy specimen. Electron microscopic findings: Myelinated nerve fibers were extensively destroyed. Wallerian degeneration could be observed (white star), and Schwann cells and macrophages were not observed. Scale bars: 2μm.

Methylprednisolone (60 mg Q.D.) was administrated as starting dose with subsequent tapering. After glucocorticoid treatment, her main symptoms were relieved, and inflammatory markers rapidly decreased, but the paresthesia and decreased sensation in her extremities decreased muscle strength with MRC (Medical Research Council) grades 2–3, tendon reflexes diminished to the disappearance, and imbalance progressed remarkably so that she was not able to walk or write; even words started slurring in the third week. We ordered an MRI of the spine with no positive findings, excluding the possibility of the patient's lumbosacral ganglion compression. Heavy metal and toxicants blood tests were negative. Head MRI and cerebrospinal fluid (CSF) examination were normal. The anti-ganglioside GQ1b antibody was negative in both serum and cerebrospinal fluid. Polymyositis antibodies, and paraneoplastic syndrome-related antibodies (Hu, Yo, and Ri antibodies) showed no meaningful findings. We performed the electrophysiological examination, and nerve and muscle biopsy revealed immune-mediated extensive peripheral nerve injury (Supplementary Figures 1–3). Intriguingly, electron microscopy images of nerve biopsy demonstrate the typical manifestation of distal axonal degeneration due to nerve injury—Wallerian degeneration (Figure 2). Paresthesia, muscle weakness, and tendon reflexes were fully recovered with MRC grades five after 9 months of glucocorticoid therapy in the patient, and slight numbness of the fingers and toes remained. She got fully recovered after more than 3 years of regular follow-up.

## Discussion

Neurological complications of AOSD are rare (2), usually seizures, cranial nerve palsy, aseptic meningoencephalitis, and Miller-Fisher syndrome, of which immune-mediated peripheral nerve injury has been reported minimally. We summarized previous case reports of AOSD combined with peripheral nerve injuries in Table 1. The delayed onset of peripheral nerve injury involving medium and large myelinated sensory fibers occurred 2 weeks to 3 years after the onset of the early symptoms, which was a great challenge for early identification and accurate diagnosis.

**Table 1:** Summary of literature on adult-onset Still's disease^*^ with peripheral nerve injury.

**Author**,							
**year of publication**	**Case characteristics**						
	**Gender/ age**	**Clinical symptoms**	**Peripheral nerve lesion and the time of onset**	**Electrophysiological examination and pathological biopsy**	**CSF examination**	**Treatment**	**Prognosis/follow-up**
Marie (3)/1999	M/17	High fever, sore throat, myalgia, headache	Left facial nerve palsy and weakened tendon reflex occurred three weeks after onset	NA	White blood cell count 25 × 10^6^/L, Protein quantitative 1 g/L	aspirin 3 g/d	All symptoms disappeared after anti-inflammatory treatment / no recurrence after seven months of follow-up
Desai (4)/2002	F/23	Joint pain, rash, fever	Miller-Fisher syndrome occurred three years after the onset, and tendon reflexes were weakened	Prolonged F wave latency of right tibial nerve	Cell count is negative; protein quantification is 0.41 g/L	Intravenous injection of immunoglobulin,2 mg/kg*d, for three days	After one week of immunotherapy, Miller-Fisher syndrome disappeared, and there was still persistent fatigue. /The follow-up results were not reported.
Zhao (5)/2008	M/60	High fever, joint pain	Five months after the onset of illness, bilateral limb weakness	Sensory nerve conduction velocity slows down, and sural nerve biopsy shows axonal damage with mild demyelination changes	No positive findings	Intravenous maintenance dose of prednisone, 20 mg/d	The right muscle weakness disappeared after eight days of hormone shock/All symptoms disappeared after four months of follow-up.
LI (6)/2009	F/62	High fever, joint pain, skin sheal	Numbness and pain in both lower limbs occurred more than two weeks after the onset	Electromyography suggests peripheral nerve damage in both lower limbs	NA	Low-dose glucocorticoids and tripterygium glycosides, etc.	After hormone and immunosuppressive treatment, the skin lesions, fever, joint pain, and skin lesions subsided, and the remaining symptoms were numbness of the lower limbs. No further follow-up was reported.

* All the cases are in line with Yamaguchi criteria. CSF, cerebrospinal fluid.

We confirmed the patient's severe immune-mediated sensory neuropathy by electrophysiological examination and pathological findings from nerve and muscle biopsies. For etiologies of sensory nerve injury, sensory ganglionopathy should be ruled out. After detailed examinations and long-term follow-up observation, we excluded the common causes of sensory ganglionopathies, such as lumbosacral ganglion compression, paraneoplastic sensory ganglionopathy, and sensory ganglionopathy secondary to Sjögren disease.

Guillain-Barré syndrome was the disease we focused on differential diagnosis in the diagnosis of sensory neuropathy in this patient. Although we found that this patient had a number of inconsistencies with the common Guillain-Barré syndrome and its common variants, for example, we found no obvious antecedent events such as infection, vaccination, and other predisposing factors such as drugs, surgery, radiation, chemotherapy, etc. that contributed to the Guillain-Barré syndrome. The patient had normal CSF cell count and protein levels, negative for anti-ganglioside GQ1b antibody, and the patient's response to glucocorticoid therapy was good. However, based on the results of the nerve conduction study and nerve and muscle pathology, theoretically, we cannot in an absolute sense rule out the possibility of the Guillain-Barre syndrome, since both Guillain-Barre syndrome and sensory neuropathy secondary to AOSD are peripheral nervous system damage due to autoimmune abnormalities. From the perspective of disease “monism,” combined with the patient's symptoms, auxiliary examination and treatment response, and long-term regular follow-up monitoring, we currently tend to believe that the patient's severe sensory neuropathy is secondary to AOSD.

Although the pathogenesis of sensory neuropathy and AOSD is unclear, the possible mechanism of its peripheral nerve damage may be related to both inflammatory response and autoimmune response. The enhanced inflammatory factors may directly attack the dorsal root ganglion, anterior horn motor neurons, interstitial structure, myelin, and axons, or infringement of nerve nourishing blood vessels cause peripheral nerve lesions (7). The autoimmune response can directly cause disease or facilitate potential pathogenic conditions, resulting in peripheral nerve lesions similar to the Guillain-Barré syndrome. Although AOSD diagnosis requires exclusion of infection, it has also been suggested that one of the possible pathogenesis of peripheral nerve injury is triggered by multiple infectious factors in an autoimmune background. They may become potentially invasive antigens to trigger peripheral neuroimmune damage, which causes the immune recognition system to misjudge and trigger autoimmune attacks. The delayed aggravation of peripheral nerve injury symptoms is consistent with this kind of autoimmune injury characterized by a prodromal period (8).

Most previous case reports lack pathological evidence; only one of the case reports showed axonal damage with mild demyelination changes by nerve biopsy. For the first time, we report typical Wallerian degeneration in AOSD patients with sensory neuropathy by neuromuscular biopsy. Wallerian degeneration is a common pathological feature of neurological disorders. It has been said that Wallerian degeneration is an active and dynamic cellular process that provides a chance for axons to reconnect, rather than rebuilding the entire nerve projection by axon regeneration, which was regulated at molecular and cellular levels (9). In response to immune-mediated injury, Wallerian degeneration showed as an orchestrated interplay between innate-immune cells and molecules produced by immune and non-immune cells.

Previous studies reported that activation of innate immunity plays a major role in the pathogenesis of AOSD, and innate immunity is also proven to contribute to the mechanism of Wallerian degeneration. All the symptoms, including that of peripheral nerve injury, were recovered by long-term glucocorticoid therapy. Delayed aggravation of the patient's peripheral nervous system symptoms may be due to Wallerian degeneration and dynamic self-destruction progress, which is a response to active systemic immunity in AOSD. Effective innate immune response to Wallerian degeneration after the injury is key to neurological recovery.

Since reports of sensory neuropathy secondary to AOSD are rare, there is currently no unified treatment recommendation. The patient started immunosuppressive therapy for AOSD. After a diagnosis of severe sensory neuropathy, we recommended glucocorticoids in combination with cyclophosphamide (CTX), but the patient refused because of concerns about the side effects of CTX. Due to economic reasons, the patient refused to use intravenous immunoglobulin (IVIG) and plasma exchange therapy and maintained glucocorticoids alone, followed by long-term tapering and eventually got remission. Combined with the above immunopathological mechanisms of AOSD and Wallerian degeneration, we believe that immunotherapy, in addition to glucocorticoids, such as glucocorticoids combined with other immunosuppressive agents, IVIG, or plasma exchange, may improve patient outcomes. Therefore, attention to neurological symptoms, early diagnosis, and initiation of adequate immunosuppressive therapy is critical to improving prognosis in patients with such severe sensory neuropathy.

## Conclusion

In conclusion, we present the rare case of a 20-year-old woman who was diagnosed with AOSD with severe sensory neuropathy. For the first time, we report typical Wallerian degeneration found in the patient by neuromuscular biopsy. By elucidating possible autoimmune mechanisms and reviewing previous reports, we conclude that Wallerian degeneration is the innate immune response to AOSD peripheral nerve injury, which may cause the delayed aggravation of neurological symptoms during glucocorticoid treatment in an AOSD patient. We emphasize that intensive attention to neurological symptoms, administration of adequate and appropriate prolonged immunosuppressive therapy, and long-term follow-up are particularly important for these patients.

## Data availability statement

The raw data supporting the conclusions of this article will be made available by the authors, without undue reservation.

## Ethics statement

Written informed consent was obtained from the individual(s) for the publication of any potentially identifiable images or data included in this article.

## Author contributions

HC wrote this case report, including the concept of this article, the definition of intellectual content, and data acquisition. HL guided the microphotograph identification of the patient's sural nerve biopsy specimens and the discussion on sensory neuropathy. YW and G-QW designed and reviewed the manuscript for its intellectual content. YW is the principal investigator. HC, HL, and G-QW are the main participants in the project. All authors contributed to the article and approved the submitted version.

## Funding

This work was supported by the National Natural Science Foundation of China (#82070605) and the Science and Technology Department of Xinjiang Uygur Autonomous Region (#2020A03004-3).

## Conflict of interest

The authors declare that the research was conducted in the absence of any commercial or financial relationships that could be construed as a potential conflict of interest.

## Publisher's note

All claims expressed in this article are solely those of the authors and do not necessarily represent those of their affiliated organizations, or those of the publisher, the editors and the reviewers. Any product that may be evaluated in this article, or claim that may be made by its manufacturer, is not guaranteed or endorsed by the publisher.
