# Supplementary material for: Case report: Wallerian degeneration: The innate-immune response to adult-onset Still's disease peripheral nerve injury
Source: Front Neurol. 2022 Oct 19;13:1016393. doi: 10.3389/fneur.2022.1016393 (PMC9626822; doi:10.3389/fneur.2022.1016393)
Supplement: Supplementary file 1 [file Data_Sheet_1.pdf]

MCV	DL-Left (ms)	Amp-Left (mv)	CV-Left (m/s)	CD-Left (mm)	DL-Right (ms)	Amp-Right (mv)	CV-Right (m/s)	CD-Right (mm)
**Medianus**								
Wrist-APB	2.52	7.8		50.0	2.98	11.8		60.0
Elbow-Wrist	6.56	6.7	54.5	220	6.44	11.8	57.8	200
**Peroneus**								
Ankle-EDB	3.50	4.7		60.0	3.35	4.8		60.0
Fib.head-Ankle	10.7	4.4	42.4	305	10.2	5.2	43.8	300
**Ulnaris**								
Wrist-ADM	1.77	10.7		55.0	1.88	12.6		55.0
Bl. elbow-Wrist	5.42	10.6	50.7	185	4.92	13.8	55.9	170

SCV	DL-Left (ms)	Amp-Left (μv)	CV-Left (m/s)	CD-Left (mm)	DL-Right (ms)	Amp-Right (μv)	CV-Right (m/s)	CD-Right (mm)
**Medianus**								
Dig III-Wrist	NE	NE			NE	NE		
**Peroneus superfic**								
Calf-Med.Dor.Cutan.	NE	NE			NE	NE		
**Radialis**								
EPL tendon-Wrist	NE	NE			NE	NE		
**Suralis**								
Mid.lower leg-Lat.Malleolus	NE	NE			NE	NE		
**Tibia Pos-2**								
Plantar med-MM	NE	NE			NE	NE		
**Ulnaris**								
Dig V-Wrist	NE	NE			NE	NE		

F-wave	FCV (m/s)	CD (mm)	Mean F-M latency (ms)	F occurrence rate (%)	M Latency (ms)
**F-Ulnaris**					
Wrist-ADM	50.6	620	25.5	100%	1.95

H-reflex	H latency (Left)(ms)	H/M Amp ratio(Left)	M Latency (Left)(ms)	H latency (Right)(ms)	H/M Amp ratio(Right)	M Latency (Right)(ms)
**H-Tibialis**						
Knee-Soleus	NE	NE	4.6	NE	NE	3.7

Supplementary Figure 1. The patient's nerve conduction study(NCS) revealed extensive peripheral nerve injury and sensory nerve predominantly. The bilateral median nerve, bilateral ulnar nerve, bilateral radial nerve, bilateral sural nerve, bilateral superficial peroneal nerve, and bilateral posterior tibial sensory nerve conduction evoked potential were not evoked. The motor conduction velocities of the bilateral median nerve, bilateral ulnar nerve, and bilateral common peroneal nerve were normal. The F-wave conduction velocity of the left ulnar nerve was normal. Both tibial nerve H-reflexes were not evoked.

MCV: Motor conduction velocity, SCV: sensory conduction velocity, DL: distal latency, Amp: amplitude, CV: conduction velocity, CD: conduction distance, APB: abductor pollicis brevis, EDB: extensor digitorum brevis, ADM: abductor digiti minimi, NE: not evoked, FCV: F wave conduction velocity.
