# Supplementary material for: Case report: Wallerian degeneration: The innate-immune response to adult-onset Still's disease peripheral nerve injury
Source: Front Neurol. 2022 Oct 19;13:1016393. doi: 10.3389/fneur.2022.1016393 (PMC9626822; doi:10.3389/fneur.2022.1016393)
Supplement: Supplementary file 2 [file Data_Sheet_2.pdf]

SEP 1 Up 3ch

Recording Site		Latency	
		Left(ms)	Right(ms)
Erb's	N9	9.0	10.9
	N9'	6.8	9.2
C4'-Fz/C3'-Fz	P14	19.4	NE
	N20	22.7	NE
	P25	30.6	33.3
	N35	38.1	38.4
	P45	53.3	48.4
	P14'	18.0	
	N70	65.8	59.0
Amplitude		Left (µV)	Right (µV)
Erb's:N9- Erb's:N9'		5.6	2.7
C4'-Fz:P14-C4'-Fz:P14'		0.26	NE
C4'-Fz:N20-C4'-Fz:P14		0.29	NE
C4'-Fz:P25-C4'-Fz:N20		0.47	NE
C3'-Fz:N35-C3'-Fz:P25		0.68	2.9
C3'-Fz:P45-C3'-Fz:N35		1.45	2.0
C3'-Fz:N70-C3'-Fz:P45		1.46	5.5

SEP 2 low 2ch

Recording Site		Latency	
		Left(ms)	Right(ms)
Popliteal(PF)	N9	10.1	9.7
	N9'	9.1	8.6
C4'-Fz/C3'-Fz	P30	43.9	NE
	P38	53.0	NE
	P60	66.8	NE
	P30'	42.0	NE
	P38'	46.6	NE
	P60'	60.6	NE
Amplitude		Left (µV)	Right (µV)
PF:N9- PF:N9'		0.20	1.00
C4'-Fz:P30-C4'-Fz:P30'		0.60	NE
C4'-Fz:P38-C4'-Fz:P38'		1.30	NE
C4'-Fz:P60-C4'-Fz:P60'		0.20	NE

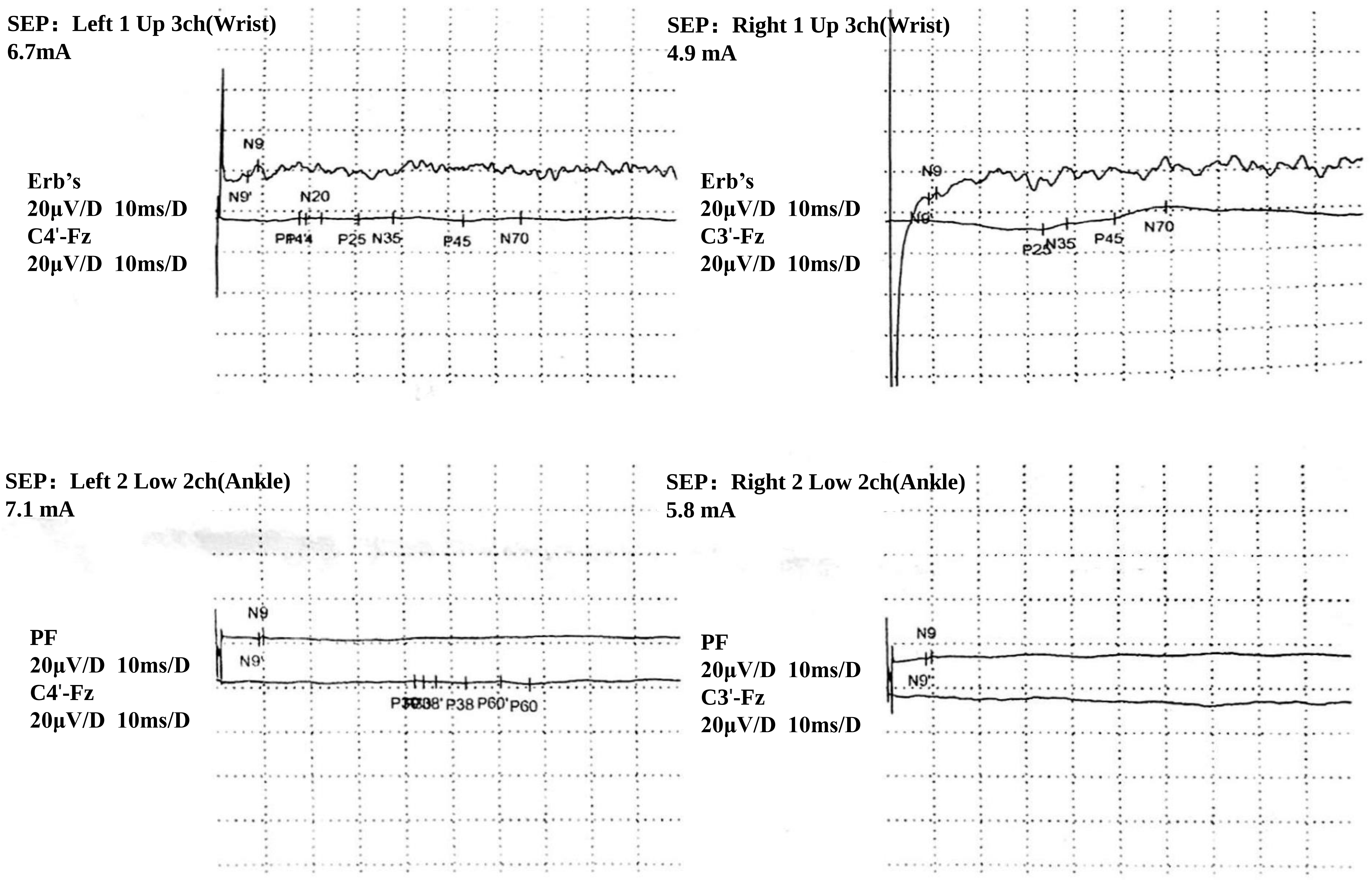

Supplementary Figure 2. Somatosensory evoked potentials(SEP) suggest sensory neuropathy/ganglionopathy.

Bilateral median nerve stimulation, Erb's point, left and right central recording, somatosensory evoked potential: right median nerve stimulation P14, N20 was not evoked, and the rest were normal. Left median nerve stimulation P14, N20 latency was prolonged, and the rest were normal. Bilateral posterior tibial nerve stimulation, popliteal fossa, left and right central recordings, and somatosensory evoked potentials: P30 and P38 were not evoked by right posterior tibial nerve stimulation, and the N9 latency was normal. Left posterior tibial nerve stimulation P30, P38 latency was prolonged, N9 latency normal. NE: not evoked.
