# Supplementary material for: Case report: Wallerian degeneration: The innate-immune response to adult-onset Still's disease peripheral nerve injury
Source: Front Neurol. 2022 Oct 19;13:1016393. doi: 10.3389/fneur.2022.1016393 (PMC9626822; doi:10.3389/fneur.2022.1016393)
Supplement: Supplementary file 3 [file Data_Sheet_3.pdf]

Neuropathological examination and diagnostic report
**Muscle pathological diagnosis:**<br>mild neurogenic pathological changes in skeletal muscle (left gastrocnemius muscle)
**Neuropathological diagnosis:**<br>severe active axonal peripheral neuropathology (left gastrocnemius muscle)
**Commentary**: The main pathological changes in skeletal muscle are two types of small horn-like atrophic muscle fibers, which are distributed in groups, and the muscle fibers tend to be grouped, which is consistent with the pathological changes of mild neurogenic skeletal muscle damage. A few intermuscular capillaries were heavily stained with NSE, and the muscle fibers had a diffuse and weak positive expression of MHC-1. Individual non-necrotic sarcolemma and a small amount of complement deposition in individual intermuscular capillaries suggested accompanying immune abnormalities. The main pathological changes of peripheral nerves are moderate to severe loss of nerve fibers and more axonal degeneration, consistent with the pathological changes of severe active axonal peripheral neuropathy.

Supplementary Figure 3. Neuropathological examination and diagnostic report.

NSE: neuron-specific enolase, MHC-1: major histocompatibility complex 1.
